# Virtual Supervision of Third Year Medical Students Using Handheld POCUS Devices and Cloud-based Image Archiving Provides Opportunity for Feedback and Skill Improvement

**DOI:** 10.24908/pocus.v8i1.16195

**Published:** 2023-04-26

**Authors:** Sydney Murray, Krista Trinder, Linden Kolbenson, Jeremy Katulka, Paul Olszynski

**Affiliations:** 1 College of Medicine, University of Saskatchewan Saskatoon, SK Canada; 2 Department of Medicine, University of Saskatchewan Saskatoon, SK Canada; 3 Department of Emergency Medicine, University of Saskatchewan Saskatoon, SK Canada

**Keywords:** Point of Care Ultrasound, Butterfly iQ, undergraduate clerkship, Image Review

## Abstract

**Background** **: **Feedback on Point of Care Ultrasound (POCUS) skills is essential for skill development. Providing feedback can be difficult in a large province with several distributed medical education sites. Use of handheld POCUS devices and a cloud-based image archiving enables virtual supervision. We evaluated the quality of uploaded images as well as feedback provided to students. **Methods: **Volunteer third year students were given access to handheld POCUS devices at various training sites. Students were encouraged to upload educational POCUS scans to their accounts where they would then receive feedback from faculty. Subsequently, images that met inclusion criteria were randomized and reviewed by a blinded expert using a global rating scale. Feedback was also analyzed. Finally, students completed a questionnaire on their technology-enhanced POCUS learning experience. **Results: **An independent-sampled t-test comparing mean ratings for initial images submitted prior to any feedback with those submitted after three rounds of feedback showed significant effect on image scores (2.60 vs 3.50, p = .040, d = .93). Feedback included 4 performance domains (indications, image generation, interpretation, and integration). Students found the technology easy to use and felt feedback was tailored to their learning needs.** Conclusions: **We observed that virtual feedback provided to medical students through a cloud-based work platform can be effective for enhancing POCUS skills.

## Introduction

Clinicians use Point of Care Ultrasound (POCUS) at the bedside to improve diagnostic accuracy and procedural safety, thus improving quality of care 1, 2. As the number of POCUS applications continues to grow, requests for foundational POCUS training in undergraduate medical education (UGME) have also increased 2, 3, 4. As early as 2014, half of Canadian medical schools had implemented some form of POCUS education into their UGME programming 5. In a recent survey of all medical schools in the USA, 69 out of 122 responding schools (57%) reported implementation of POCUS training at the UGME level 6.

At the University of Saskatchewan, POCUS is integrated across all 4 years of UGME. In pre-clerkship, learning is facilitated in the clinical skills courses through a flipped-classroom model that includes video tutorials and hands-on scanning sessions with simulated patients for a total of 10 hours of supervised POCUS practice/student. After an introduction to POCUS fundamentals, students are shown how and when to scan for ascites, pleural effusion, pericardial effusion, hydronephrosis and bladder volume 7. These POCUS applications are integrated in the Clinical Skills curriculum and have been included in student assessment through assignments and Objective Structured Clinical Examination (OSCE) stations. During their clerkship years, students are encouraged (though not required) to further develop their POCUS skills as they rotate through their core clinical rotations. This is supported by additional online POCUS modules and quizzes in some core rotations. They may also apply to the POCUS elective offered during their final year of clerkship 8. 

Surveys on POCUS integration in UGME consistently rank the lack of qualified instructors as a main barrier to implementation 5, 6. The distribution of trainees across several geographically distinct training sites over the course of their medical training further compounds this challenge. The above significantly limits opportunities for directly supervised scanning during core clinical rotations. Obtaining supervision and feedback over the duration of training are essential to POCUS skill development 9, 10, 11, 12.

With these challenges in mind, the University of Saskatchewan’s College of Medicine purchased and deployed 20 handheld POCUS devices to be used by students during their clinical rotations in clerkship at the College’s many training sites. Through cloud-based image archiving and review, as well as tele-guided POCUS instruction, this pilot program enables supervision of scans continues regardless of where students are posted. The aim of this study was to determine if virtual POCUS supervision resulted in improved POCUS skills amongst clerkship trainees.

## Methods

### Setting/Study Participants

This study was reviewed by the University of Saskatchewan’s Research Ethics Board and granted exemption status as it was deemed consistent with program evaluation (Beh ID 3343). A total of 11 3^rd^ year students had access to the POCUS handheld devices (ButterflyNetwork, Inc. New York, USA) across several training sites (cities of Regina, Saskatoon, Prince Albert, and Swift Current) for the duration of the study period. Students signed-out devices for several weeks, during which time they were expected to practice the POCUS applications introduced to them in the pre-clerkship curriculum. Trainees were encouraged to upload anonymized scans to the cloud-based archive. Uploaded scans were regularly reviewed by authors PO and LK. Feedback on uploaded scans was provided to trainees through the image archiving platform. 

### Blinded Image Review 

To determine if POCUS skills were improving, scans uploaded by trainees between February to June of 2022 were selected and downloaded from the platform. POCUS scans/applications that were within the taught curriculum were chosen for blinded review, totaling 91 scans submitted by 9 students. Scans were assigned a numerical code prior to blinding and randomization. Author JK then evaluated each scan using a checklist and global rating scale adapted from the American College of Emergency Physicians Emergency Ultrasound Standard Reporting Guidelines 13. The scale includes a scoring range from 1 (no recognizable structures, no objective data can be gathered) to 5 (Minimal criteria met for diagnosis, all structures imaged with excellent image quality and diagnosis completely supported).

### Analysis of Written Feedback 

We also examined several aspects of the feedback provided to trainees over the study period including time from image upload to feedback from faculty, the word count of the feedback for a given scan/image, the nature of feedback, and whether a virtual dialogue occurred between faculty reviewer and trainee. The type of written feedback was categorized as it related to 4 POCUS performance domains: image generation, image optimization, clinical integration, and knowledge of indications 10.

### Participant Questionnaire

We developed a questionnaire based on a framework used for evaluating technology-enhanced learning materials^14^. The questionnaire uses both a 7-point Likert scale and includes both affirmative and negating stems, as well as free-text short-answer questions. The questionnaire was distributed to all participants who had signed-out the handheld devices over the study period. Four of the 11 students completed the questionnaire. 

### Tele-guidance POCUS Session Questionnaire

Students were also provided opportunities to take part in tele-guided POCUS instruction using a feature associated with the handheld device’s image archiving platform. Teleguided POCUS instruction by the study team included prompting students to perform trouble-shooting maneuvers to optimize image generation as well as offering feedback on core transducer techniques. As above, we developed a modified questionnaire used for evaluating technology-enhanced learning materials^14^. This was distributed to participants after each teleguided POCUS session. Two of the 3 students who took part in a tele-guidance session completed the tele-guidance questionnaire.

### Anonymity

All information regarding learners identity/training was kept confidential and not disclosed to the blinded reviewer. No identifiable patient information was included in any of the images. All questionaries were anonymous. Data was reported in aggregate form so that it is not possible to identify individuals.

### Blinded image review

In total, 91 images submitted by 9 students met inclusion for blinded review, with each student submitting multiple images on separate occasions. Data were coded based on whether an image was submitted before students received any feedback (N = 35), if they received feedback once (N = 30), if feedback was received twice (N = 16), or if prior feedback was received on three separate occasions (N = 6). Using SPSS software, analyses of variance (ANOVA) were conducted comparing ratings based on the number of times students received feedback to determine if there was a difference overall. Post-hoc t-tests were also conducted to compare whether significant differences were found based on the number of times feedback was received.

### Analysis of assessment feedback and questionnaires.

All information was tabulated in an Excel spreadsheet. Averages and standard deviations were calculated for: time the feedback was given from the time the trainee uploaded the scan, the word count of the feedback, and the type of feedback. Questionnaire responses (Likert scores) were tabulated in an Excel spreadsheet. Means and standard deviations were calculated.

## Results

### Blinded image review

**Figure 1:**
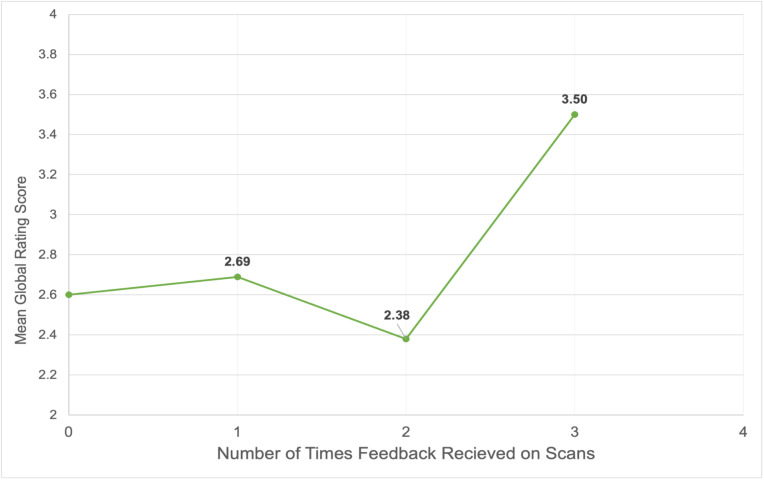
Comparison of number of times feedback was received and mean global rating scores.

An ANOVA comparing global ratings based on the number of times feedback was received was not statistically significant (p = .160). However, an independent-samples t-test, comparing the mean ratings for initial images submitted with no feedback with those submitted after feedback had been received three times showed a significant difference (t(39) = 2.12, p = .040, d = .93). Cohen’s d values equal or greater than 0.8 mean a large effect (Table 1, Figure 1). 

**Table 1:** Comparison of global rating scores based on number of times feedback was received. An ANOVA comparing global ratings based on the number of times feedback was received was not statistically significant (p = .160). However, an independent-samples t-test comparing the mean ratings for initial images submitted with no feedback with those submitted after feedback had been received three times showed a significant difference (t(39) = 2.12, p = .040, d = .93). This difference had a large effect size.

**Number of Times Feedback Received**	**Mean**	**Standard Deviation**	**N**
0	2.60	.98	35
1	2.69	1.23	29
2	2.38	.81	16
3	3.50	.84	6

### Analysis of Assessment Feedback 

**Figure 2:**
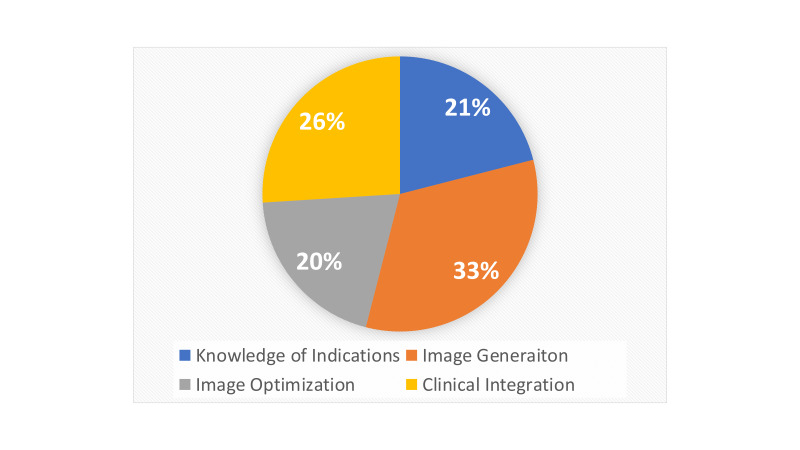
Breakdown of feedback provided by faculty reviewers on uploaded trainee scans.

A total of 107 scans were uploaded by the trainees (16 scans were outside the POCUS curriculum and thus not included in the blind image analysis and included obstetrical scans and procedural guidance), and 80 out of scans received feedback from the reviewers, for a feedback responses rate of 74.77%. Average time from upload to initial feedback was 2 days 8 hours (+/- 4 days). Average word count for feedback was 36.99 ± 25.57 words. Feedback from 72 out of 80 scans fell within the 4 performance subcategories (Figure 2). There were several instances of feedback including more than one performance domain 10. 

### Participant Questionnaire Responses

Mean scores reveal that participants felt that the POCUS handheld device program was well organized (M = 6.00, SD = .82), the technology was easy to navigate (M = 6.75, SD = .50), and the feedback provided on images was helpful in their ultrasound skill development (M = 6.75, SD = .50). Overall, the responses of the questionnaire indicated that learners would prefer to sign out handheld POCUS devices for a period from 4-6 weeks, with three of the four respondents selecting this option. Student comments indicated that they found the feedback provided by physicians trained in POCUS was helpful and helped them improve their performance. Two students indicated that they were not always comfortable with their scans being visible to other users on the cloud-based archive.

### Tele-guidance Session Questionnaire

The tele-guidance sessions were roughly 30-35 minutes in duration. Likert scores reveal that students who took part in the sessions felt the tele-guidance session was tailored to their learning needs and improved their POCUS skills. Students commented that the one-on-one feedback contributed to their learning.

## Discussion

We observed that virtual feedback provided to medical students through a cloud-based work platform can be effective for enhancing POCUS skills. An independent-sampled t-test comparing mean ratings for initial images submitted prior to any feedback with those submitted after three rounds of feedback showed significant effect on image scores (2.60 vs 3.50). Feedback included 4 performance domains (indications, image generation, interpretation, and integration). Students found the technology easy to use and felt feedback was tailored to their learning needs.

Direct supervision of scanning combined with regular image review have long been the backbone of postgraduate POCUS training in Emergency Medicine 15. Our findings suggest that image review can also be used to support undergraduate POCUS training. By the time they have reached clerkship, most students would have performed 10-15 scans/application under the supervision of our clinical skills instructors. Their scans, and associated image scores, underscore the very gradual progression of POCUS skills amongst most trainees, consistent with previous studies on POCUS learning curves for POCUS skills 9, 10, 11, 12. When asked whether feedback received on images was helpful, students indicated strong agreement. This supports our view that virtual feedback provided to medical students creates opportunities to enhance trainees’ POCUS skills.

The teleguided POCUS instruction questionnaire responses indicated some interesting themes. Student responses indicated that the tele-guided sessions were tailored to their learning needs and that their learning needs were met. Moreover, tele-guided sessions were reported to improve POCUS skills. With respect to the challenges of geographical location, the promising results from the tele-guided instruction questionnaire suggest that efforts to provide virtual POCUS teaching in such a technology-enhanced manner are worthwhile.

The main limitation of this study is the small sample size of students and inherent selection bias associated with trainees volunteering for such a training opportunity. This was partially countered by the fact that trainees performed several scans, thus providing a reasonable sample for blinded image analysis (91 images). Similarly, due to the smaller number images uploaded after three rounds of feedback and a single blinded reviewer, statistical results should be interpreted with caution. A larger sample size would allow for repeated measures ANOVA that would measure ratings matched across students rather than at an aggregate level. Lastly, though we are not aware of students receiving any direct feedback or supervision from other faculty during the study interval, it is possible some skill development also occurred this way. 

## Conclusions

We observed that regular feedback provided to medical students through a cloud-based work platform was associated with improved POCUS images. Our findings are consistent with previous studies that have shown gradual learning curves of ultrasound skills and techniques when applying hands-on practice.We intend to expand our handheld POCUS device program and continue to evaluate its effectiveness at supporting student learning.

## Statement of ethics approval/consent

Ethical approval for this study was granted by the University of Saskatchewan Research Ethics Office (REB# Beh3343).

## Funding

This study was funded by the University of Saskatchewan’s College of Medicine Dean’s Summer Research Projects Fund.

## Conflicts of Interest

PO served on the CanHealth Advisory Board for Point of Care Ultrasound. 
